# Editorial: Microbial remediation of heavy metal pollution

**DOI:** 10.3389/fmicb.2026.1887178

**Published:** 2026-06-15

**Authors:** Iftikhar Ahmed, Alejandro Rodriguez-Sanchez, Duraipandiyan Veeramuthu, Pankaj Kumar Arora

**Affiliations:** 1 National Culture Collection of Pakistan (NCCP), Land Resources Research Institute (LRRI), National Agricultural Research Centre (NARC), Islamabad, Pakistan; 2 Department of Microbiology, University of Granada, Granada, Spain; 3 Division of Microbiology, Entomology Research Institute, Loyola College, Chennai, India; 4 Department of Plant Science, Mahatma Jyotiba Phule Rohilkhand University, Bareilly, India

**Keywords:** bioaccumulation, bioremediation mechanisms, biosorption, environmental sustainability, heavy metals, microbial remediation, plant–microbe interactions

Heavy metals pollution is an increasing global problem affecting the ecosystem, agriculture, and public health. Heavy metals are non-biodegradable and persist for several years in the soil and water, where they can persist for decades and enter the food chain through plant uptake and trophic transfer (Priya et al., 2022). Metals such as cadmium (Cd), lead (Pb), chromium (Cr), arsenic (As), and mercury (Hg) are particularly problematic due to their toxicity, mobility, and capacity to disrupt cellular processes, including enzyme activity, membrane integrity, and genetic stability (Abbas et al., 2015a, 2014, 2015b; Manzoor et al., 2019; Mustapha et al., 2026). The traditional remediation methods which include chemical stabilization, treatment by adsorption, and soil excavation are not very effective. Also, they are very expensive and energy intensive and may create other pollution problems (Mohamed et al., 2025). These limitations have driven increasing interest in biologically based solutions that are both sustainable and environmentally compatible.

**Figure 1:**
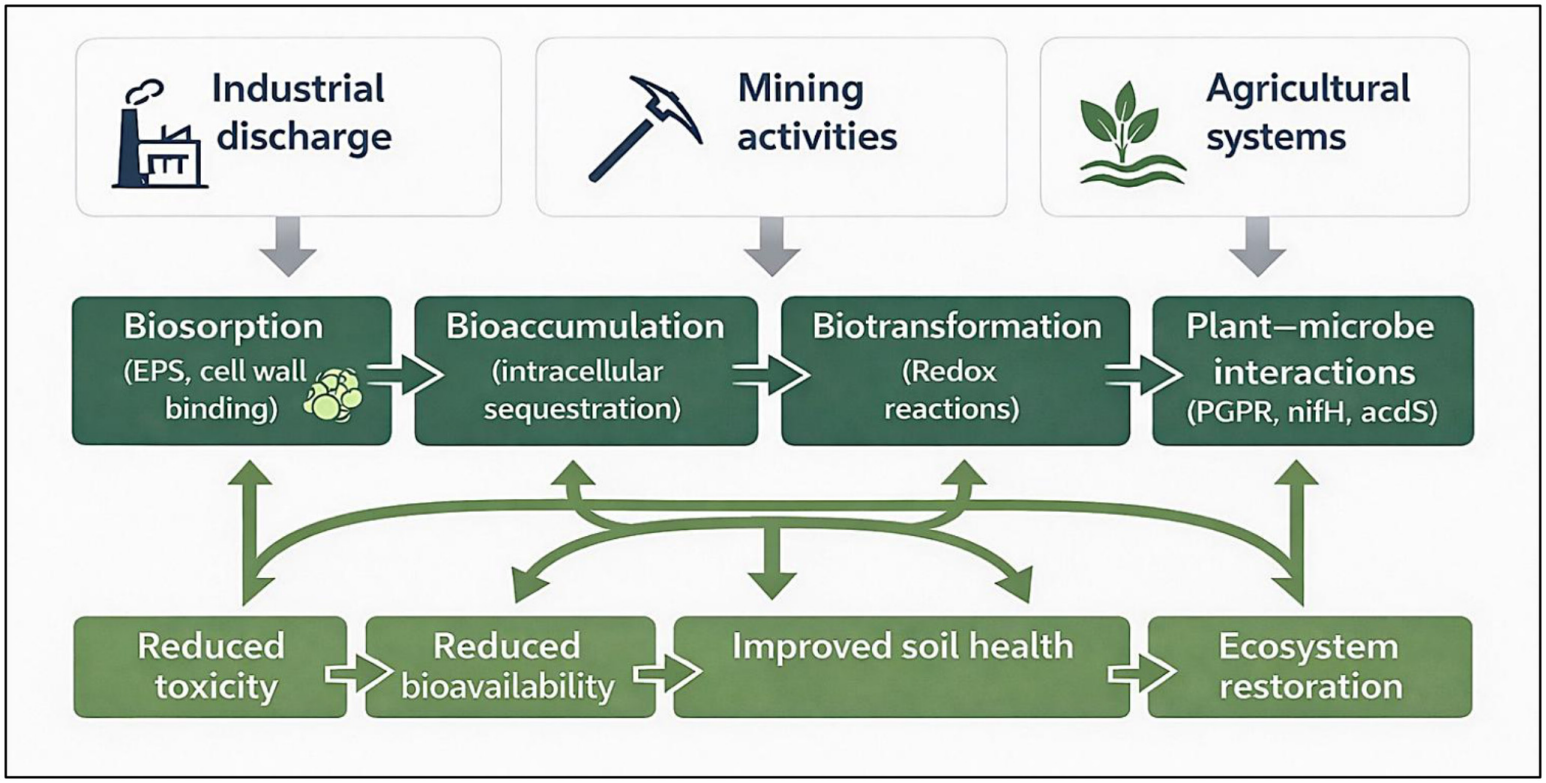
Conceptual framework of microbial mechanisms for heavy metal remediation.

Microorganisms play a vital role in the removal of heavy metals from the environment through various mechanisms (Naz et al., 2016). The major microbial mechanisms involved in heavy metal remediation are biosorption, bioaccumulation, biotransformation, biomineralization, and bioleaching, which contribute to the reduction of heavy metal pollution in the environment (Chugh et al., 2022). The use of microorganisms to remediate pollution is an exciting new alternative to traditional methods. The unique metabolism of microorganisms can be harnessed to either preserve heavy metals in a non-toxic state or alter and sequester heavy metals (Abo-Alkasem et al., 2023; Dhakal et al., 2025). The new disciplines of genomics, synthetic biology, and molecular biology have given new ways to engineer and create microorganisms with abilities to enhance heavy metal remediation and improve the efficiency of treatments in polluted regions. The present Research Topic aims to enhance our knowledge of microbial processes involved in heavy metal remediation and to identify new, innovative, and more sustainable approaches to restore contaminated environments. The Research Topic consists of 13 contributions, including original research, reviews, and opinion pieces, which provide a thorough overview of microbial bioremediation in different environments. The key microbial mechanisms involved in heavy metal remediation are summarized in Figure 1.

Caldeira et al. reviewed some of the recent literature on the response of bacteria to the presence of the environmentally relevant metal, vanadium, that has not been extensively studied. This paper analyzed the dynamics of oxidative stress, metal resistance, and mechanisms such as denitrification, and proposed that the occurrence of vanadium could potentially be relevant to the processes of biogeochemical cycles. The authors pointed out important gaps in the literature, including poorly characterized vanadium-specific reductases and vanadium transport systems.

In this manner, a systematic review of *Pseudomonas* spp. and heavy metal bioremediation was carried out by De Santis et al. using PRISMA. Their study revealed unprecedented genetic and metabolic features that enable this genus to adapt to multi-metal and fluctuating environmental conditions through efflux pumps, redox enzymes, EPS formation, and biofilm-mediated resilience. Experimental research presented in this Research Topic offers comprehensive insights into microbial processes involved in metal immobilization and detoxification. Maumela et al. showed that extracellular polymeric substances (EPS)-producing *Bacillus* spp. could be optimized to maximize lead biosorption whereby by inclusion of a hydrophobic fraction lead removal efficiencies were >90%. Moreover, this research showed that it is possible to recover and reuse the biosorption materials, thereby enhancing sustainability in technologies that utilize this type of biosorption. Similarly, Zhang et al. demonstrated that EPS-producing bacterial strains can alter heavy metal speciation in soil aggregates, thereby promoting the formation of stable mineral phases such as carbonates and phosphates. These changes reduced cadmium and lead uptake in pakchoi by up to 80%, highlighting the potential of microbial interventions to enhance crop safety in contaminated soils.Mineral transformations through a microbial pathway can also be exemplified by the study of Li et al. that produced the first known manganese-oxidizing bacterium, which formed biogenic manganese oxides with sophisticated nanostructures and surfaces exhibiting a high reactivity. The high surface reactivity and complex nanostructures of these biogenic oxides contributed to exceptional cadmium removal efficiency (>99.5%) during the treatment of cadmium-contaminated wastewater. Zhu et al. expanded this concept to complex contamination conditions by examining biogenic sulfate-reducing bacteria growing on antimony tailings and demonstrated the effective immobilization of contaminants such as antimony, arsenic, lead, cadmium, copper, and zinc through the formation of stable sulfides and carbonates. This paper demonstrates that the significance of using microbial consortia in the management of multi-element contamination in mining-affected habitats is significant.

Bioremediation of heavy metals using microbial consortium has also been studied. Gupta and Arunachalam developed a yeast consortium, consisting of native strains isolated from the river Cauvery. These strains are highly resistant to heavy metal and used to bioremediate lead. The consortium successfully removed various concentrations of lead when introduced to alginate-based bioaugmented filtration systems, thereby proving to be scalable and usable in wastewater treatment.

The heavy metal biotransformation along with chemotactic responses has also been investigated. Garg et al. studied adaptations of microorganisms under hexavalent chromium stress using *Bacillus licheniformis* KNP. They not only reported efficient Cr(VI) reduction but also observed negative chemotaxis away from high chromium concentrations, suggesting that such movement may support bacterial survival and sustained bioremediation activity under chromium stress.With all this, the merging of microbial remediation with sustainability in agriculture is another important theme of this Research Topic. For example, Abbas et al. isolated and characterized 68 heavy metal-tolerant bacterial strains from industrial discharge-contaminated environments, demonstrating high tolerance levels to multiple metals, including chromium, copper, cadmium, and arsenic. Biosorption assays revealed strong removal capacities, particularly for lead and cadmium, with several isolates showing high efficiency. Molecular characterization indicated that the strains belonged to diverse genera, including *Bacillus, Pseudomonas*, and *Staphylococcus*, reflecting broad functional diversity. In addition, the detection of plant growth-promoting genes such as *nifH* and *acdS* highlighted their potential role in nitrogen fixation and stress alleviation. Greenhouse experiments further confirmed that selected strains enhanced the growth of *Brassica napus* under heavy metal stress. Together, these findings demonstrate the potential of these isolates as multifunctional candidates for integrated bioremediation and sustainable agricultural applications. In a complementary study, Kaushal and Pati reviewed the role of plant growth-promoting rhizobacteria (PGPR) in heavy metal detoxification, emphasizing their ability to improve nutrient availability, modulate phytohormone levels, and mitigate oxidative stress in plants growing in contaminated soils.

Guan et al., explored integrated remediation strategies combining microbial and physicochemical treatments to evaluate the effectiveness of minerals and compost in lead-contaminated paddy soils. This experiment resulted in decreases of lead bioavailability and uptake by plants and an enhancement of soil microbial diversity and enzymes, which demonstrates incomplete recovery of soil ecological processes. In addition to experimental and review studies, this Research Topic also includes forward-looking perspectives and critical analyses. Hui proposed an innovative bioremediation strategy involving genetically engineered microalgae for the bioadsorption and enzymatic transformation of mercury species, including the highly toxic methylmercury. Although there is a significant potential in this innovation, there are serious biosafety, genetic stability, and ecological concerns. Shi et al. also emphasized the need for more stringent methodologies in microbial remediation studies, highlighting issues such as limited taxonomic resolution associated with 16S rRNA analysis, inadequate reporting of pollutant levels, and insufficient biosafety evaluation prior to outdoor experimental applications.

Taken together, the research papers published in this Research Topic show the extraordinary diversity, versatility and functional capacity of microbial assemblages to heavy metal contamination. They emphasized that microbial remediation is a complex process involving interconnected physicochemical and biological mechanisms operating across multiple spatial and temporal scales. Simultaneously, these contributions indicate certain issues, such as the necessity to have common methodology, better reproducibility, and translatability of laboratory results to field facility implementation.

In conclusion, this Research Topic presents a synthesis of innovations in the field of remediation of heavy metal pollution by microbes in an impressive way and at the right time. Through its combination of mechanistic understanding and practical initiatives, it will help to create sustainable and scalable solutions to environmental restoration. Further directions in future studies should emphasize on the field validation, merging multi-omics techniques to dissect complicated interactions among microbes, and the production of safe and regulation friendly microbial technologies to stabilize their adequate application in actual environmental management practices.
